# TP53 Staining in Tissue Samples of Chronic Lymphocytic Lymphoma Cases: An Immunohistochemical Survey of 51 Cases

**DOI:** 10.4274/tjh.2016.0115

**Published:** 2017-03-01

**Authors:** İbrahim Kulaç, Çetin Demir, Yahya Büyükaşık, Tezer Kutluk, Ayşegül Üner

**Affiliations:** 1 Hacettepe University Faculty of Medicine, Department of Pathology, Ankara, Turkey; 2 Hacettepe University Faculty of Medicine, Department of Pediatrics, Division of Pediatric Oncology, Drug Resistance Laboratory, Ankara, Turkey; 3 Hacettepe University Faculty of Medicine, Department of Internal Medicine, Division of Hematology, Ankara, Turkey; 4 Author is currently appointed at Johns Hopkins University Faculty of Medicine, Baltimore, USA

**Keywords:** TP53, Immunohistochemistry, Chronic lymphocytic lymphoma, Proliferation centers

## Abstract

**Objective:**

Chronic lymphocytic leukemia (CLL) is the most common lymphoproliferative disease in adults. The aim of this study is to find out if the extent of proliferation centers or the immunohistochemical expression of p53 is related to disease prognosis.

**Materials and Methods:**

In the scope of this study, 54 biopsy specimens from 51 patients (50 of lymph nodes; the others of spleen, tonsil, orbit, and liver) diagnosed with CLL at the Hacettepe University Department of Pathology in 2000-2013 were reevaluated. The clinical and demographic data of the patients were obtained from our patient database. Biopsy samples were assessed semi-quantitatively for the percentage of proliferation center/total biopsy area (PC/TBA) and an immunohistochemical study was performed on representative blocks of tissues for p53 expression.

**Results:**

When the patients were divided into two categories according to Rai stage as high and low (stages 0, 1, and 2 vs. stages 3 and 4), it was seen that patients with low Rai stage had a better prognosis than those with high stages (p=0.030). However, there was no statistically significant correlation between overall survival and PC/TBA ratio or p53 expression levels.

**Conclusion:**

In our cohort, PC/TBA ratio and immunopositivity of p53 did not show correlations with overall survival.

## Abstract (tr)

**Amaç:**

Kronik lenfositik lösemi (KLL) erişkin bireylerde en sık görülen lenfoproliferatif hastalıktır. Bu çalışmanın amacı KLL tanısı almış hastaların doku örneklerindeki proliferasyon merkezlerinin yaygınlığı ve p53 ekspresyonu ile prognozları arasında bağlantı olup olmadığını araştırmaktır.

**Gereç ve Yöntemler:**

Bu çalışma kapsamında Hacettepe Üniversitesi Tıp Fakültesi Hastanesi Patoloji Anabilim Dalı’nda 2000-2013 yılları arasında KLL tanısı almış 51 hastanın 54 biyopsi örneği yeniden değerlendirilmiştir. Hastaların klinik ve demografik verileri hasta veri tabanından elde edilmiştir. Yapılan incelemede biyopsi örneklerinde proliferasyon merkezlerinin tüm biyopsi alanına oranı (PM/TBA) yarı niceleyici olarak değerlendirilmiş ve seçilen temsili bloklardan elde edilen kesitlerde immünohistokimyasal yöntemle p53 ekspresyonuna bakılmıştır.

**Bulgular:**

Hastalar Rai evrelerine göre düşük ve yüksek olmak üzere iki gruba ayrıldığında düşük evreli hastaların genel sağkalım sürelerinin yüksek evreli hastalara göre daha uzun olduğu görülmüştür (p=0,030). Ancak, proliferasyon merkezi oranı veya p53 ekspresyon düzeyleri arasında istatistiksel olarak anlamlı ilişki gösterilememiştir.

**Sonuç:**

Çalışmamıza dahil edilen hasta grubunda PM/TBA oranı ve p53 immünpozitifliginin sağkalım ile ilişkisi olmadığı görülmüştür.

## INTRODUCTION

**Figure 1:**
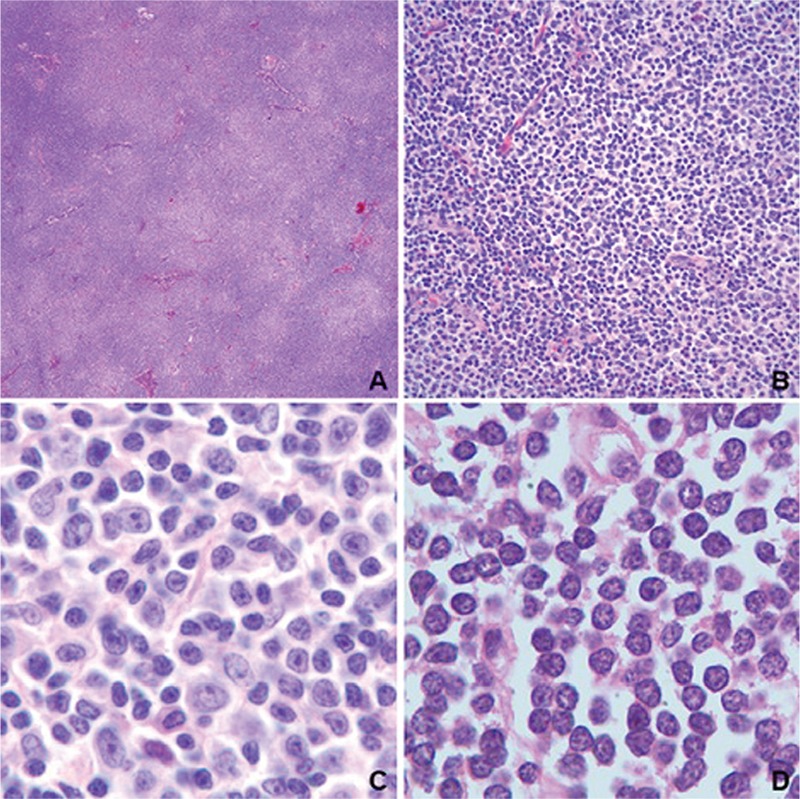
Representative images of a lymph node with chronic lymphocytic leukemia involvement. On low power, lightly colored areas represent proliferation centers (A). Proliferation centers are rich in prolymphocytic cells (C). Other areas are predominantly composed of small lymphocytic cells (B and D).

Chronic lymphocytic leukemia (CLL) is the most common lymphoproliferative disorder of adults in Western countries [1]. A vast majority of the patients present at Rai stage 0 or 1, most incidentally diagnosed during routine blood work-up [2]. Although lymph node biopsy is not a standard first-line diagnostic tool, in some instances such as transformation or an unexpected clinical course, it is indicated. In hematoxylin and eosin-stained sections of involved lymph nodes it is characterized by a diffuse infiltrate of small uniform cells with occasional segregation of relatively larger cells (Figure 1). It is well known that a large percentage of patients will be followed for a long time without progression, but some will eventually progress and require treatment. For decades several different parameters have been used to identify patients with a predisposition to progression. lymphocyte doubling time or several biochemical and flow cytometry-based markers are widely used for this purpose [3,4,5,6]. Though these are still important, novel molecular tests offer a wide range of highly impactful markers [7]. Several chromosomal abnormalities have also been shown as considerable prognosticators, such as del13q14, trisomy12, del11q22-23, and del17p13, as well as translocations that include 14q32 and are the most commonly used ones in routine hematology/pathology practice [8,9].

Mutations on the short arm of chromosome 17 have also been studied extensively and shown as some of the most important cytogenetic alterations associated with adverse prognosis [10,11,12]. Patients with 17p13 deletion tend to have the worst outcome compared to patients with any other cytogenetic or mutational anomalies and they also have a better response to certain types of treatments [13,14]. The 17p13 locus harbors *TP53*, one of the most important genes in cancer pathogenesis. The presence of *TP53 *alterations in CLL patients has been reported at between 7% and 33% in the literature [14,15,16,17]. Immunohistochemical methods, by using anti-p53 antibody, have been utilized for detection of p53 alterations for years. Although interpretation varies in different tumor types, it is highly reliable as a surrogate marker. However, there are no studies on immunohistochemical assessment of p53 alteration using formalin-fixed paraffin-embedded (FFPE) solid organ samples of CLL patients.

In this study, our aim was to identify the frequency and the effect on overall survival (OS) of *TP53* abnormalities in FFPE tissue samples in a cohort of 51 patients from a single institution, and we also evaluated the impact of some clinical parameters on clinical outcome.

## MATERIALS AND METHODS

Fifty-four solid organ biopsies from 51 patients, which were evaluated between 2000 and 2013 at the Hacettepe University Department of Pathology, were included in the study. Patients’ date of initial diagnosis, date of the biopsy procedure, follow-up time, date of death if applicable, and platelet, leukocyte, hemoglobin, lactate dehydrogenase, and absolute lymphocyte levels were recorded.

Because of potential decalcification artifacts and the paucity of neoplastic cells in bone marrow samples, we decided to use samples from solid organ biopsies (especially lymph nodes), which are larger in size and free of decalcification artifacts.

### Morphological Evaluation

All the biopsy samples were reevaluated by two pathologists (A.Ü., İ.K.). The percentage of the area of proliferation centers (PCs) was determined semi-quantitatively for each biopsy sample using hematoxylin and eosin-stained slides after selecting the most representative slide. Tru-Cut biopsies and liver and spleen biopsy samples were excluded from this particular scoring.

### Immunohistochemical Studies

**Figure 2:**
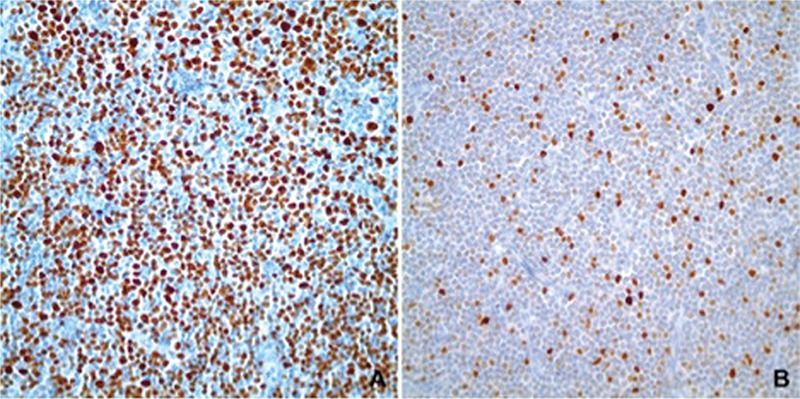
p53 expression detected by immunohistochemistry: A) a case with >90% p53 positivity; B) a case with 10% p53 positivity.

For the detection of p53 protein in samples, immunohistochemical studies were performed on sections of 5 µm obtained from one representative block from each sample. All stainings were done automatically by using anti-p53 antibody (ScyTek, Logan, UT, USA; Clone: DO-7, Lot: 23081) and the iView^TM^ DAB Detection Kit (Ventana, Tucson, AZ, USA) on the Ventana Benchmark XT platform. Figure 2 demonstrates p53 staining of two representative cases.

The p53 staining was scored semi-quantitatively as the percentage of nuclear-positive cells among all cells by selecting 10 random areas on each slide, counting at least 500 cells in each selected area, and calculating the mean of each individual value.

### Statistical Analysis

Numeric variables were analyzed by their mean and minimum-maximum values, while categorical variables were included in analysis as numbers and percentages. Categorical and continuous data were compared by the chi-square test (or Fisher exact test if required by sample size) and Mann-Whitney U test, respectively. The Spearman correlation coefficient was used for the comparison of two numeric variables. OS was calculated from diagnosis to the date of mortality of any reason. The patients who did not die were censored at the last follow-up for OS computation. Survival analyses were computed by the Kaplan-Meier method. Comparisons of survival rates were done by log-rank test. The statistical significance threshold was accepted as p<0.05. SPSS 15 (SPSS Inc., Chicago, IL, USA) was used for statistical analyses.

## RESULTS

### Clinical Findings

Thirty-five (68.6%) of the patients were male and 16 (31.4%) were female. Mean overall follow-up time was 48.4 months (range: 1-135 months). Mean age of the patients at the time of diagnosis was 60.3 years (range: 41-83). At the time of the study, 11 patients were deceased due to events related to CLL. At the time of diagnosis, 4 patients were at Rai stage 0, 21 patients were at Rai stage 1, 5 patients were at Rai stage 2, 2 patients were at Rai stage 3, and 10 patients were at Rai stage 4. Due to a lack of clinical data we were not able to assess the Rai stage of 9 cases.

**Figure 3:**
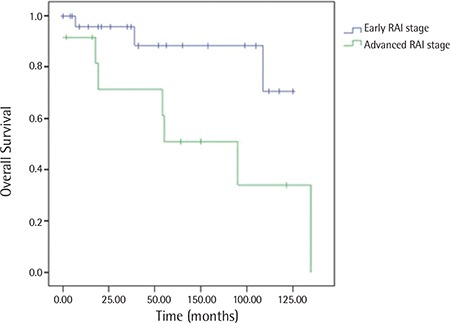
Kaplan-Meier graph of the survival of patients grouped by Rai stages.

Patients were divided into two groups according to their Rai stages at the time of diagnosis (“low stage” defined as Rai 0, 1, and 2; “high stage” defined as Rai 3 and 4) and survival analysis was performed between these two groups. As shown in Figure 3, patients with low Rai stages had better OS than patients with high Rai stages (p=0.030).

### Morphological and Immunohistochemical Findings

Fifty of the 54 samples were from lymph node biopsies and the rest were from tonsil, liver, orbit, or spleen. Samples were evaluated and the percentages of the area of PCs were recorded using hematoxylin and eosin slides. The percentage of PCs as a continuous variable did not seem to have a relationship with the death rate. Another survival analysis was performed after dividing patients into two groups using a cut-off of 40%. Although there seemed to be a trend for a relatively better outcome for patients with more prevalent PCs, this difference was not statistically significant (log rank, χ^2^=3.82, p=0.0508).

**Figure 4:**
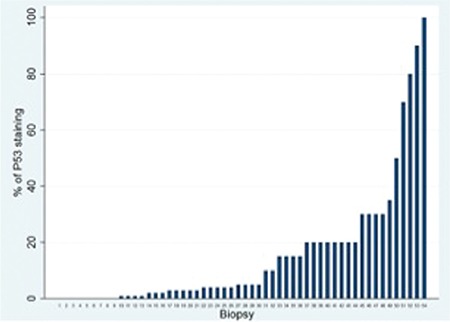
Distribution of p53 expression through biopsy samples.

**Figure 5:**
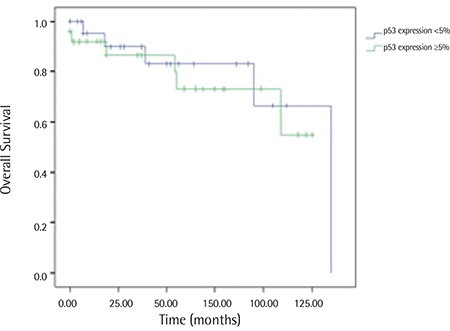
Kaplan-Meier graph of the survival of patients grouped by p53 staining.

Distribution of percentages of p53 staining is shown in Figure 4 (n=54). Correlation analysis between p53 expression and OS showed no statistically significant result (Spearman’s rho=0.55, p=0.701). Patients were also grouped into two categories according to their p53 scores, using the median value, as ≤5% and >5%. Survival analysis between these two groups did not show a statistically significant difference, as displayed in Figure 5 (log rank, χ^2^=0.08, p=0.7771).

Five of the biopsies showed focal prolymphocytic transformation; while two of these biopsies showed no p53 staining, the other three had p53 scores of 15%, 60%, and 100%. Because the number of biopsies with transformation was small, statistical analysis could not be performed to evaluate the correlation of p53 alteration and prolymphocytic transformation.

## DISCUSSION

CLL is a substantial health problem for the entire world, but specifically for developed countries as it is mainly a disease of the elderly. With the increase of overall life expectancy the number of CLL patients will increase accordingly. Although the vast majority of CLL patients remain in a dormant state, some will rapidly (and sometimes unexpectedly) progress and display an aggressive course. Some prognostic factors have been proposed over the years; among them, alterations in the *TP53* gene (including cytogenetic alterations in chromosome 17) stand out as those with the most impact.

Among clinical parameters, Rai staging seems to be a reliable and consistent indicator for CLL patients, and in our study, we also showed that advanced Rai stage is associated with a worse disease outcome.

PCs in CLL are predominantly composed of larger cells with open chromatin, larger nuclei, and relatively prominent nucleoli. For years, researchers believed that the extent of PCs was associated with worse prognosis since PCs are metabolically and mitotically active zones. So far, however, only a limited number of studies showed findings that supported this hypothesis [18], while others usually failed to demonstrate a correlation between the extent of PCs and prognosis [19,20,21]. In our study, we also could not show a significant correlation. Larger cohorts are needed to clarify this issue.

Studies in various tumor types showed variable patterns of p53 staining that correlated with alterations in *TP53* at the genomic level. In one study, it was reported that >5% of p53 staining in hepatocellular carcinoma is a reliable surrogate marker for *TP53* gene alterations [22]. In a different study published in 2011, Yemelyanova et al. showed that diffuse positivity of p53 staining in ovarian carcinoma cases is highly correlated with *TP53* mutation [23]. Thus, setting a threshold for p53 positivity and enabling immunohistochemistry to reflect *TP53* mutation requires a high number of cases and a more detailed analysis, but our sample size was not sufficient for such a comprehensive work-up.

There are limited numbers of studies focusing on the immunohistochemical evaluation of p53 and clinical outcome in CLL patients. Schlette et al. showed that an extent of 40% or more p53 staining in bone marrow samples of CLL patients correlated with shorter survival [24]. One study by Cordone et al. showed that p53 positivity correlated with a shorter treatment-free interval using neoplastic lymphocytes obtained from peripheral blood and setting a cut-off of 1% of the lymphoid cells [25]. In our study, however, we could not show any significant correlation between p53 immunopositivity in the involved solid organ samples that could be due to problems in specimen handling, tissue fixation, and/or processing, or in short all the steps of the pre-analytic phase of a biopsy. Another possible explanation is that solid organ samples are not as representative as circulating neoplastic cells in the peripheral circulation. Neoplastic cells, which reside in lymph nodes, might represent only a subset of the entire CLL population.

In this study we also made a morphological observation that we thought could be interesting. The p53 positivity was stronger and more prevalent in prolymphocytic cells within the PCs compared to small lymphocytic cells. Although this raises the possibility that initial alterations might be originating from the PCs, this observation needs further investigation.

There are some weaknesses of our study. We did the p53 scoring semi-quantitatively, although quantitative analysis using image analyzer software would provide a more precise evaluation of p53 expression. Another important point is that a sequence analysis of the p53 gene in the samples could be very helpful for correlation with p53 staining. Our samples were FFPE tissues and some were more than 10 years old, which makes obtaining a good quality of DNA almost impossible.

## CONCLUSION

In conclusion, our study is one of the first studies that aimed to assess the impact of p53 staining in solid organ biopsy samples and overall survival in CLL patients. Contrary to the well-confirmed prognostic value of del17p in FISH analysis, *TP53* immunohistochemistry does not have a similar impact. This finding should be confirmed with larger series.
